# Impact of Childhood Maltreatment on Recidivism in Youth Offenders: A Matched-Control Study

**DOI:** 10.1177/0093854815598598

**Published:** 2015-10

**Authors:** Dongdong Li, Chi Meng Chu, Joseph Teck Ling Goh, Irene Y. H. Ng, Gerald Zeng

**Affiliations:** Ministry of Social and Family Development; National University of Singapore; Ministry of Social and Family Development

**Keywords:** childhood maltreatment, family violence, neglect, risk assessment, propensity score matching, YLS/CMI

## Abstract

The purpose of the study is to examine the impact of childhood maltreatment on youth offender recidivism in Singapore. The study used case file coding on a sample of 3,744 youth offenders, among whom about 6% had a childhood maltreatment history. The results showed that the Youth Level of Service/Case Management Inventory 2.0 (YLS/CMI 2.0) ratings significantly predicted recidivism for nonmaltreated youth offenders, but not for maltreated youth offenders. Using propensity score matching, the result from a Cox regression analysis showed that maltreated youth offenders were 1.38 times as likely as their nonmaltreated counterparts to reoffend with a follow-up period of up to 7.4 years. The results implied that the YLS/CMI 2.0 measures were insufficient for assessing the risk for recidivism for the maltreated youth offenders, and that other information is needed to help assessors use the professional override when making the overall risk ratings.

According to the World Health Organization (WHO), childhood maltreatment includes

all forms of physical and/or emotional ill-treatment, sexual abuse, neglect or negligent treatment or commercial or other exploitation, resulting in actual or potential harm to the child’s health, survival, development or dignity in the context of a relationship of responsibility, trust or power. (Butchart, Harvey, Mian, & Furniss, 2006, p. 9)

It is a social problem in many societies, and has been associated with detrimental long-term effects on educational achievement, mental and physical health, as well as behavioral difficulties (Behl, Conyngham, & May, 2003; Fergusson, Boden, & Horwood, 2008; Gilbert et al., 2009). Disturbingly, one of these detrimental outcomes of child maltreatment is that the victims are likely to exhibit behavioral difficulties and subsequently become perpetrators of crime (e.g., Godinet, Li, & Berg, 2014; Keiley, Howe, Dodge, Bates, & Pettit, 2001; Kelley, Thornberry, & Smith, 1997). This vicious cycle has been supported in many empirical studies (Bender, 2010; Widom, 1989).

## Childhood Maltreatment and Subsequent Delinquency

Across many studies, childhood maltreatment has been shown to consistently predict subsequent behavioral difficulties and delinquency in early adolescence (Godinet et al., 2014; Keiley et al., 2001; F. Li & Godinet, 2014; R. Thompson & Tabone, 2010). Research in Western contexts has demonstrated that children with a history of childhood maltreatment have a much higher risk of being arrested and/or referred for delinquent offenses (Fagan, 2005; Jonson-Reid, 2002; Kelley et al., 1997; Lemmon, 1999; Swanston et al., 2003; Widom & Maxfield, 2001). In a large-scale study of 18,676 children and youth, Ryan and Testa (2005) found that the delinquency rates for substantiated victims of childhood maltreatment were on average 47% higher than those who were not maltreated. In addition, a longitudinal study of 1,575 individuals showed that maltreated children were significantly more likely to be arrested as a juvenile (27.4% vs. 17.2%) and adult (41.6% vs. 13.9%) when compared with their nonmaltreated counterparts (Widom & Maxfield, 2001). Children who have experienced maltreatment were also more likely to commit offenses as adults (Fagan, 2005; Mersky & Topitzes, 2010), become delinquent at a younger age (Lemmon, 1999; Ryan, Herz, Hernandez, & Marshall, 2007), and commit a violent offense (Kelley et al., 1997; Widom & Maxfield, 2001).

Viewing the issue from another perspective regarding the experience with maltreatment among youth offenders, the extant literature also suggests that a substantial proportion of youth offenders are likely to have a history of child maltreatment. In a study of childhood maltreatment prevalence across public service sectors, 77.6% of youth from the juvenile justice sample (*n* = 229) had reported being maltreated, with 57.3% having experienced multiple forms of maltreatment (Miller, Green, Fettes, & Aarons, 2011). King and colleagues (2011) showed that 65% to 75% of youth offenders (*n* = 1,735) reported having experienced physical abuse, whereas 10% to 40% were reportedly sexual abused during their childhood. Furthermore, Moore, Gaskin, and Indig (2013) found that 60% of young offenders have a history of childhood abuse or neglect, with females being almost 10 times as likely to report three or more types of serious child maltreatment compared with males. In fact, the prevalence of childhood maltreatment among juvenile delinquent population is substantially greater than that in the general population (Wiebush, Freitag, & Baird, 2001).

## Childhood Maltreatment and Recidivism

Considering that childhood maltreatment is strongly associated with delinquency, there are strong grounds to expect childhood maltreatment to also associate with recidivism. Previous research showed that the rate of recidivism was higher for youth offenders with childhood maltreatment history. A study on Arizona administrative data revealed that those dually involved cases were twice as likely to recidivate (62% vs. 30%) as compared with delinquency only cases (Halemba, Siegel, Lord, & Zawacki, 2004). In a more recent study on youth who simultaneously received services from the child welfare and juvenile justice agencies (*n* = 1,148), the rate of recidivism more than 5 years for these maltreated and nonmaltreated offenders was 56% and 41%, respectively (Huang, Ryan, & Herz, 2012). Similarly, Ryan (2006) demonstrated that youth offenders (*n* = 286) with a history of childhood physical abuse and neglect were 1.58 times more likely to recidivate within 10 years (50% vs. 37%) as compared with youth offenders who were not abused.

This link is also supported in two meta-analyses with a small, but significant effect sizes between childhood maltreatment and youth offender recidivism. Cottle, Lee, and Heilbrun (2001) demonstrated that childhood maltreatment was a significant predictor of recidivism among youth offenders, notwithstanding that its effect size is weaker than many other risk factors (*n* = 9,949, *k* = 5). In another meta-analysis with 1,542 sexually abusive adolescents, Mallie, Viljoen, Mordell, Spice, and Roesch (2011) also found that there is a significant (albeit small) relationship between history of childhood sexual abuse and sexual recidivism (odds ratio = 1.51, *p* < .05) from 29 effect sizes that were obtained from 11 studies.

Despite the link between childhood maltreatment and juvenile offender recidivism, few studies have examined the differences in the background characteristics between juvenile offenders with and without childhood maltreatment. It is well documented that both childhood maltreatment and youth offending behaviors might be preceded by a common set of risk factors, such as family environment (Margolin & Gordis, 2000; Repetti, Taylor, & Seeman, 2002). Without controlling for the possible background differences, it is impossible to conclude that childhood maltreatment history is a unique contributor to the continuation of the delinquent behavior. In other words, it is difficult to judge how much of the variations in recidivism risk is due to childhood maltreatment or the background characteristics without a proper control group (Widom, 1989).

## Risk Assessment and Classification

The higher rate of recidivism among youth offenders with childhood maltreatment history further highlighted the importance of risk assessment of these youth. The Risk–Need–Responsivity (RNR) framework (Andrews & Bonta, 2010) states that effective offender rehabilitation requires the accurate classification of the offender’s level of risk and needs. Furthermore, the framework states that intervention should target those criminogenic needs that are functionally related to criminal behavior; and equally important, the style and mode of intervention should match the offender’s abilities and learning style.

One of the most widely used products of the RNR principles is the Youth Level of Service/Case Management Inventory (YLS/CMI; Hoge & Andrews, 2002, 2011). The YLS/CMI 2.0 is a structured assessment instrument designed to facilitate the effective intervention and rehabilitation of youth who have committed criminal offenses (aged 12-18 years) by assessing their risk level, criminogenic needs, and strengths. The YLS/CMI 2.0 assesses the level of risk based on eight types of risk factors. However, childhood maltreatment as a static risk factor for recidivism was not used in assessing the risk levels, although it was included in the assessment of other needs for the purpose of case management. Empirical support for the utility of the YLS measures has been reported in many studies from both Western and Asian contexts (see Chu et al., 2015; Chu, Yu, Lee, & Zeng, 2014). However, several studies reported that the YLS measures only explained about 10% of the variation of the recidivism risk between offenders (Onifade et al., 2008) and that the measure failed to distinguish the survival rates between low and moderate risk cases (Schmidt, Campbell, & Houlding, 2011). One reason is that the YLS measures focused only on dynamic risk factors (which are amendable through interventions) and thus may not be suitable to assess long-term recidivism. In other words, the unexplained variation may be due to other factors such as the youth offenders’ environment (e.g., neighborhood crime rate) or other relatively static risk factors such as the underlying personality traits. If youth offenders with childhood maltreatment history were from different backgrounds with different risks and needs, it is logical to assume that the YLS measures will not accurately differentiate the risk levels for them.

## Assessing the Youth Offenders in Singapore

Singapore is an independent island state in South East Asia with a total population of 5.4 million (Singapore Department of Statistics, 2013). Many statutes in Singapore are based on English common law (e.g., the Criminal Procedure Code, 2012), but there are some statutes that are based on legislation from other jurisdictions; for example, the Children and Young Persons Act (2001) is based on child protection legislation in the United Kingdom and Northern Ireland. As such, there are similarities in the way that offenses are defined in Singapore when compared with the abovementioned countries, but the exact language of the laws might vary somewhat.

Cultures and societies often define what attitudes and behaviors are considered “normal” and “deviant.” Notwithstanding that there is some agreement across cultures about what constitutes offending behavior, the development of deviant attitudes and behaviors can vary due to cultural norms, gender roles, morals, religion, taboos, and expectations. Similarly, cross-cultural studies have shown that the meaning of child maltreatment varies across Western and Asian cultures (Deater-Deckard & Dodge, 1997). For example, strict discipline (such as caning) is seen as a sign of parental concern and involvement rather than child abuse in Asian societies (Elliott, Tong, & Tan, 1997; Lau, Liu, Yu, & Wong, 1999). In a recent study, D. Li, Chu, Ng, and Leong (2014) found a different developmental trend in the risk of reentry into child protection systems in Singapore as compared with Western countries. Such differences in the sociocultural context will not only change the antecedents but also moderate the effects of child maltreatment (Gershoff, 2002). It is possible that the prevalence rate of childhood maltreatment among youth offenders may differ across countries. It is also possible that the motivation, risk factors, and pathways for offending may differ due to cross-cultural differences as to how individuals cope, self-regulate, or even report crime.

The youth and adult correctional services in Singapore have adopted RNR framework to provide a theoretical and empirical-based approach to conduct offender assessment and rehabilitation since early 2000s. Importantly, the YLS/CMI (and subsequently the YLS/CMI 2.0) was chosen as the primary risk assessment measure to assess the risk and needs of youth offenders and adapted for local usage (Chua, Chu, Yim, Chong, & Teoh, 2014). A recent validation study showed that the YLS/CMI 2.0 Overall Risk Rating and Total Score were moderately predictive for general recidivism over a mean follow-up period of 1,764.5 days (Chu et al., 2015). Overall, the results suggest that the YLS/CMI 2.0 is suited for assessing youth offenders in terms of their risk for general recidivism within a non-Western context.

## The Present Study

Despite the available literature on childhood maltreatment and offending, there is a dearth of local Singaporean research looking at this unique group of youth that straddle the dual statuses of being both a victim and offender. Importantly, the extant literature on the utility of risk assessment measures for predicting recidivism in youth offenders also did not examine the impact of child maltreatment on the risk classifications, which could have far-reaching implications on sentencing, risk management, and intervention. Thus, this study sought to contribute via examining the link between childhood maltreatment and recidivism among youth offenders in Singapore. We first examined the prevalence rate of childhood maltreatment among youth offenders in Singapore. We then examined whether (a) the youth offenders with a history of childhood maltreatment were different from their counterparts without such history in terms of background characteristics; (b) YLS/CMI 2.0 accurately measures the risk of recidivism for maltreated and nonmaltreated youth offenders; and (c) childhood maltreatment was a unique contributor of recidivism in a matched-control sample.

## Method

### Sample

The sample included a total of 3,744 youth (aged 12-18 years) who were charged between January 2004 and December 2008. The sample represented 97% (3,264/3,370) of the youth offenders on community supervision and 99% (480/485) in youth correctional institutions during this period; the remaining could not be coded as a result of missing information or file retrieval difficulties. Their mean age was 15.29 years (*SD* = 1.21, *Mdn* = 15, see Table 2). Majority of the offenders were males (*n* = 3,327, 89%) and were of Chinese ethnicity (*n* = 1,938, 52%). In addition, two thirds (*n* = 2,458, 66%) of the sample committed nonviolent nonsexual offenses (e.g., theft, burglary, and substance use offenses), followed by violent but nonsexual offenses such as causing hurt and robbery (*n* = 1,198, 32%). A small group of the sample (*n* = 88, 2%) committed sexual related offenses (e.g., rape, molestation, and voyeuristic offenses).

### Definitions

#### Childhood Maltreatment

In line with a broad definition as used by WHO (Butchart et al., 2006), “childhood maltreatment” in this study was defined as having experienced or witnessed abuse or family violence. A youth offender was coded as having been maltreated if the case files had documented one of the following conditions including physical abuse, sexual abuse, neglect, or family violence. For ease of reference, these youth offenders will be referred to as “maltreated youth offenders,” and the youth offenders without such childhood maltreatment experiences as “nonmaltreated youth offenders.”

#### Recidivism

Recidivism refers to (a) any conviction of sexual (e.g., indecent exposure, molestation, peeping, rape, and sodomy), violent (e.g., physical assault, rioting, murder, and robbery), or nonviolent nonsexual (e.g., theft, fraud, burglary, drug use, and drug trafficking) offenses that were committed following the initial court order; (b) breaches of court orders; or (c) any combination of the aforementioned outcomes.

### Measure

#### Risk of Recidivism

The risk of recidivism was measured using the YLS/CMI 2.0 (Hoge & Andrews, 2011). It consists of 42 items (scored as either Present or Absent) that are divided into eight subscales (Prior or Current Offenses/Dispositions, Family Circumstances/Parenting, Education/Employment, Peer Relations, Substance Abuse, Leisure/Recreation, Personality/Behavior, and Attitudes/Orientation). The item scores (i.e., the number of indicated risk factors/needs) can be aggregated to obtain a Total Risk Score (hereafter YLS Score). The predictive validity of YLS/CMI 2.0 ratings was tested in Singapore with fine-tuned cutoff scores (Chu et al., 2015). Based on these cutoff scores, the youth offenders were categorized into low risk, moderate risk, high risk, and very high risk ratings (hereafter YLS Ratings). As the proportion of the youth offenders who were assessed as high and very high risk were small in this sample (*n* = 150, 4.0%), these two categories were grouped together for purpose of analyses.

#### Outcome Measure

The outcome variable in this study was “time to recidivism.” It was computed as the number of days from the date of the initial court order to the date of recidivism. Any youth offender without a record on recidivism by the end of the data extraction period (April 20, 2011) was coded as a “null” case and the time variable was the number of days between the date of the initial court order and the end of the data extraction period.

#### Background Variables

A total of 15 background variables, which were commonly viewed as predictors of both childhood maltreatment and offending behaviors in the literature, were coded. These background variables were grouped into three broad areas: (a) the youth offender’s personal characteristics, which include the age at the start of the order, gender, race, the presence of developmental delay, and the presence of an attention deficit hyperactivity disorder (ADHD) diagnosis; (b) household environmental characteristics, which include the presence of financial or accommodation problems, the presence of significant family trauma, whether the biological family was intact, and the presence of marital conflict; as well as (c) parental characteristics, which include father criminality, mother criminality, parents’ chronic history of offenses, parents’ emotional or psychiatric distress, parents’ drug or alcohol abuse, and whether the parents were uncooperative. These variables were coded under the assessment of other needs and special considerations in YLS/CMI 2.0. The definitions of these variables can be found in the YLS/CMI 2.0 manual (Hoge & Andrews, 2011).

### Procedure

The approval for the current research study was obtained from the Ministry of Social and Family Development. A total of eight staff, including two psychologists, one probation officer, and five research assistants, were involved in the file coding between January 2011 and September 2012. These raters had attended a 3-day training program conducted by accredited trainers; the training program involved lectures, discussions, case studies and scoring practices, as well as a test. Multiple sources of information were obtained to code for the eight subscales of YLS/CMI 2.0, the history of childhood maltreatment as well as the youth offenders’ background information. These sources of information included (a) psychological reports prepared by psychologists at the Clinical and Forensic Psychology Branch, (b) presentence reports prepared by probation officers, (c) charge sheets, (d) statement of facts, (e) any previous assessment and treatment reports, as well as (f) school reports. The coding was completed based on file information available at the time of the initial assessment at the presentencing stage; information available subsequent to the presentencing stage was not considered for coding purposes to minimize criterion contamination. After the abovementioned coding was completed, official recidivism data were obtained and the cutoff date for such data was April 20, 2011. The interrater reliability (intraclass correlation coefficients [ICCs]; single rater, absolute agreement definition) was .63 for the YLS/CMI 2.0 total score, and the ICC ranges from .43 (fair) to .60 (good) for the eight subscales and the case management inventory (see Cicchetti, 1994, for a classification of ICCs). As the study was originally designed to examine the predictive validity of the YLS/CMI 2.0 ratings in Singapore context, more detailed description of the study procedures can be found elsewhere (Chu et al., 2015; Chu et al., 2014).

### Statistical Analyses

First, we examined the prevalence rate of recidivism among maltreated and nonmaltreated youth offenders and whether they have different background characteristics using chi-square tests in the full sample using SPSS 19. Second, we tested whether the YLS/CMI 2.0 total score and Overall Risk Rating accurately measure the risk of recidivism for maltreated and nonmaltreated youth offenders using two Cox regression models. Using the YLS/CMI 2.0 total score as a continuous variable, the equation included the mean-centered YLS/CMI 2.0 total score, childhood maltreatment, and an interaction term between the two. A similar model was tested for the YLS/CMI 2.0 Overall Risk Rating as a categorical variable using the default settings of testing interaction in survival analysis where SPSS 19 automatically creates dummy coding and interaction terms in the program. As discussed in the introduction, a significant interaction was expected. Specifically, it was hypothesized that the YLS/CMI 2.0 total score and Overall Risk Rating were both predictive of time to recidivism for nonmaltreated youth offenders but not for maltreated offenders.

Third, we tested the unique contribution of childhood maltreatment to recidivism via a subsample that was matched on the 15 background variables as specified in the Method section. Propensity score matching (PSM) was conducted through the “MatchIt” module in statistical software R. PSM is a set of techniques used in nonexperimental studies to correct for selection bias and the influence of confounding variables when analyzing the causal effects of treatment (Guo, Barth, & Gibbons, 2006). PSM attempts to model the process of random assignment used in experimental studies by making the treatment group and control group equivalent in every aspect in which they were matched upon. For this study, the *childhood maltreatment history* variable functioned as the treatment condition under PSM. The 2-1 Nearest Neighbor Matching method was used because there was a relative abundance of individuals from the control group to be matched with. The balance of the matched sample was examined by comparing the distributions for all categorical covariates. After matching, chi-square tests and Cox regression analyses were conducted on the newly matched sample to compare outcomes between treatment (i.e., maltreated) and control (i.e., nonmaltreated) groups using SPSS 19. In the Cox regression analysis, all the background variables as well as the YLS/CMI 2.0 total score were included for regression adjustment to provide a “doubly robust” assessment of the treatment effect (Rubin & Thomas, 2000). Based on these procedures, any differences in outcomes between the two groups could be considered as attributable to the effect of treatment (i.e., due to having a history of childhood maltreatment) rather than any of the covariates.

## Results

### Recidivism Rate

Out of the initial sample of 3,744 youth offenders, there were 221 individuals (6%) who had a history of childhood maltreatment. For the maltreated offenders, the mean time to recidivism/end of data collection was 1,186 days (*Mdn* = 1,114, *SD* = 729). For the nonmaltreated offenders, the mean time to recidivism/end of data collection was 1,322 days (*Mdn* = 1,309, *SD* = 722). Over half of the maltreated offenders (*n* = 125, 57%) breached their current order or reoffended within the follow-up period. In contrast, the recidivism rate for the nonmaltreated youth offenders was significantly lower at 38%, *n* = 1,350; χ^2^(1, *N* = 3,744) = 28.98, *p* < .01. As shown in Table 1, the recidivism rate for maltreated offenders ranged from 20% (*n* = 45) within 1 year to 69% (*n* = 9) within 7 years. In contrast, the rate for nonmaltreated offenders ranged from 13% (*n* = 462) to 47% (*n* = 77). Without adjusting for the effects of other variables, the maltreated youth offenders were 1.62 times (95% confidence interval [CI] = [1.35, 1.94]) as likely to reoffend as compared with the nonmaltreated youth offenders as shown in a Cox regression analysis.

**Table 1:** Rate of Recidivism for Maltreated and Nonmaltreated Youth Offenders

Recidivism Within (Years)	*n*		Recidivism %		
	Total	Maltreated	Total	Maltreated	Nonmaltreated
1	3,744	221	14	20	13
2	3,744	221	24	30	24
3	3,271	195	31	42	31
4	2,458	160	37	48	36
5	1,747	112	40	59	39
6	996	65	44	59	43
7	178	13	48	69	47
Overall					
Up to 7.4	3,744	221	39	57	38

### Differences in the Background Variables

In addition to the differences in recidivism rate, the results also showed significant differences in all the background variables between maltreated and nonmaltreated offenders (Tables 2 and 3). As compared with nonmaltreated offenders, there were larger proportions of younger, female, non-Chinese, developmentally delayed, and ADHD youth among the maltreated offenders. A post hoc analysis showed that the maltreated offenders were arrested for the first time at a younger age as compared with nonmaltreated offenders, *M*_maltreated_ = 13.90; *M*_nonmaltreated_ = 14.55; *t*(3725) = 5.78, *p* < .01. There were also larger proportions of youth among the maltreated offenders that were from a background with household and parental problems, for example, 65% (*n* = 144) of the maltreated offenders versus 26% (*n* = 904) of the nonmaltreated offenders were from nonintact families. In fact, the chi-square tests were significant at *p* = .01 level for all the background variables with the exception of the diagnosis for ADHD. These results implied that children with certain characteristics were more likely to have a history of childhood maltreatment. However, as nonmaltreated and maltreated youth offenders might have different backgrounds, it was uncertain whether the differences in recidivism were due to childhood maltreatment or the background variables. Therefore, it was necessary to conduct a matched-control analysis (refer to the sections after) to rule out the possibility that any difference in recidivistic outcome was attributed to the background differences rather than childhood maltreatment.

**Table 2:** Individual Characteristics as a Percentage of the Overall Sample

	Total (*N* = 3,744)	Nonmaltreated (*n* = 3,523) %	Maltreated (*n* = 221) %
Age (years)			
12-15	2,131	56	66
16-18	1,613	44	34
Gender			
Male	3,327	90	78
Female	417	10	22
Race			
Chinese	1,938	52	44
Non-Chinese	1,806	48	56
Developmental delay			
No	3,691	99	96
Yes	53	1	4
Diagnosis of ADHD			
No	3,695	99	97
Yes	49	1	3

*Note.* ADHD = attention deficit hyperactivity disorder.

**Table 3:** Household and Parental Characteristics as a Percentage of the Overall Sample

	Total (*N* = 3,744)	Nonmaltreated (*n* = 3,523) %	Maltreated (*n* = 221) %
Financial/accommodation problems			
No	2,984	81	61
Yes	760	19	39
Significant family trauma			
No	3,582	96	89
Yes	162	4	11
Intact family			
No	1,048	26	65
Yes	2,696	74	35
Father criminality			
No	3,404	92	77
Yes	340	8	23
Mother criminality			
No	3,626	97	92
Yes	118	3	8
Parents’ chronic history of offenses			
No	3,055	83	64
Yes	689	17	36
Parents’ emotional/psychiatric distress			
No	3,673	98	94
Yes	71	2	6
Parents’ drug/alcohol abuse			
No	3,661	98	89
Yes	83	2	11
Parents’ marital conflict			
No	2,984	81	61
Yes	760	19	39
Uncooperative parents			
No	3,653	98	93
Yes	91	2	7

### Risk Assessment of Maltreated Offenders

**Figure 1:**
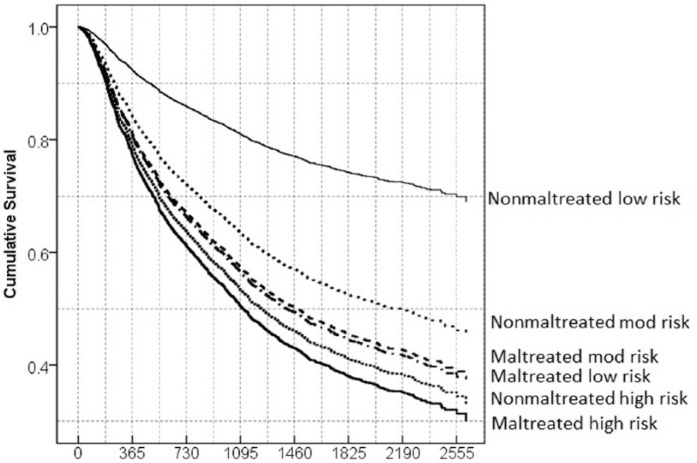
Survival curve for maltreated and nonmaltreated youth offenders with different risk levels of recidivism in the full sample

We then tested the predictive validity of YLS/CMI 2.0 total score and YLS/CMI 2.0 Overall Risk Rating in relation to recidivism for maltreated and nonmaltreated youth offenders. Table 4 presents the results of a Cox regression model with YLS/CMI 2.0 total score, childhood maltreatment, and an interaction term between the two. As expected, the results of the Cox regression analyses revealed a significant interaction effect. Further examination of the interaction effect showed that the YLS/CMI 2.0 total score was significantly related to recidivism for nonmaltreated offenders (*B* = 0.08, *SE* = 0.01, hazard ratio [HR] = 1.09, 95% CI = [1.07, 1.10]) but not for maltreated offenders (*B* = 0.02, *SE* = 0.02, HR = 1.02, 95% CI = [0.98, 1.06]). Table 5 presents the recidivism rate among offenders with different YLS/CMI 2.0 Overall Risk Ratings. The recidivism rate had a broader range from 25% (*n* = 322) to 57% (*n* = 133) for the nonmaltreated youth offenders, whereas the recidivism rate was high for all the three risk groups of maltreated youth offenders ranged from 53% (*n* = 20) to 64% (*n* = 28). Figure 1 presents the survival pattern for each group. It clearly showed that the YLS/CMI Overall Risk Rating significantly predicted recidivism for nonmaltreated youth offenders and that the hazard of recidivism for each of the three groups (Low, Moderate, and High Risk) was significantly different. In contrast, for maltreated youth offenders, the differences in the hazard of recidivism were nonsignificant among the three groups (Figure 1). Specifically, the Maltreated Low Risk youth offenders had much higher rates of recidivism compared with Nonmaltreated Low Risk offenders—53% (*n* = 20) versus 25% (*n* = 322).

**Table 4:** The Moderating Effect of Childhood Maltreatment Between YLS/CMI 2.0 Total Score and Recidivism

	*B*	*SE*	HR [95% CI]
YLS/CMI total score	0.08	0.01	1.09** [1.07, 1.10]
Childhood maltreatment	0.44	0.12	1.55** [1.24, 1.94]
Interaction (YLS/CMI total score × Childhood maltreatment)	−0.06	0.02	0.94** [0.90, 0.98]

*Note.* YLS/CMI = Youth Level of Service/Case Management Inventory; CI = confidence interval; HR = hazard ratio.

** *p* < .01.

**Table 5:** The Moderating Effect of Childhood Maltreatment Between YLS/CMI 2.0 Overall Risk Rating and Recidivism

Childhood Maltreatment	YLS/CMI 2.0 Overall Risk Rating	Total *n*	Recidivist *n*	Recidivist %	*B*	*SE*	HR [95% CI]
Nonmaltreated	Low risk	1,300	322	25			
	Moderate risk	1,984	894	45	0.77	.07	2.16** [1.90, 2.45]
	High risk	235	133	57	1.09	.10	2.97** [2.43, 3.64]
Maltreated	Low risk	38	20	53	1.00	.23	2.71** [1.72, 4.26]
	Moderate risk	137	75	55	0.97	.13	2.64** [2.05, 3.39]
	High risk	44	28	64	1.18	.20	3.24** [2.20, 4.76]

*Note.* YLS/CMI = Youth Level of Service/Case Management Inventory; CI = confidence interval; HR = hazard ratio.

** *p* < .01.

A post hoc analysis showed that Maltreated Low Risk offenders were more likely to be charged for violent offenses (*n* = 15, 40%) as compared with the Nonmaltreated Low Risk youth offenders (*n* = 341, 26%). The results implied a moderating effect of childhood maltreatment between the risk of recidivism and the event of recidivism. In other words, these results supported our hypothesis again that childhood maltreatment was a unique contributor to recidivism, and it was not well explained by the YLS/CMI 2.0 Overall Risk Ratings.

### Matched-Control Analysis

The unique contribution of childhood maltreatment to recidivism in a matched sample was examined using PSM. The maltreated offenders were matched with 442 nonmaltreated offenders, creating a new matched sample of 663 individuals. After matching, there was relatively equal distribution for all the background variables (results from the chi-square tests were all nonsignificant). The overall percentage of the maltreated youth offenders was 33% (*n* = 221) in the matched sample as compared with 6% (*n* = 221) in the original sample. The overall recidivism rate was 48% (*n* = 320) in the matched sample as compared with 39% (*n* = 1,475) of the original sample. These results indicated that the matched sample was a subset of the higher risk group from the original sample.

Table 6 presents the Cox regression results on recidivism. The results showed that maltreated youth offenders had 38% increased risk of recidivism, indicating a higher recidivism risk due to childhood maltreatment while controlling for all the background variables as well as the YLS/CMI 2.0 Overall Risk Rating. These results supported our hypothesis that childhood maltreatment history itself is a unique contributor of recidivism for youth offenders.

**Table 6:** Effect of Childhood Maltreatment on Recidivism in the Matched Sample

	*n*	Recidivist *n*	Recidivist %	*B*	*SE*	HR [95% CI]
Nonmaltreated	442	195	44.1			
Maltreated	221	125	56.6	0.32	.12	1.38** [1.10, 1.74]

*Note.* The analysis was conducted while controlling for all the 15 covariates as well as the overall YLS/CMI 2.0 Overall Risk Rating. YLS/CMI = Youth Level of Service/Case Management Inventory; CI = confidence interval; HR = hazard ratio.

** *p* < .01.

Table 7 presents the associations between the history of childhood maltreatment and the YLS/CMI 2.0 Overall Risk Rating using the matched sample. The results showed that the maltreated youth offenders were more likely to be in the High Risk category (*n* = 46, 21%) as compared with nonmaltreated youth offenders (*n* = 46, 10%). For the subscales, the results showed that the maltreated youth offenders had higher risk rating in the domains of Family Circumstances/Parenting and Personality/Behavior.

**Table 7:** Results of Chi-Square Test and Descriptive Statistics for Childhood Maltreatment by YLS/CMI 2.0 Overall Risk Rating in the Matched Sample

Maltreated	YLS Risk Level			χ^2^(*df*)
	Low	Moderate	High	
Prior and current offenses/dispositions				5.27 (2)
No	338 (77%)	102 (23%)	2 (1%)	
Yes	155 (70%)	62 (28%)	4 (2%)	
Family circumstances/parenting				39.77** (2)
No	201 (46%)	211 (48%)	30 (7%)	
Yes	57 (26%)	119 (54%)	45 (21%)	
Education/employment				2.42 (2)
No	70 (16%)	272 (62%)	100 (23%)	
Yes	31 (14%)	128 (58%)	62 (28%)	
Peer relations				5.34 (2)
No	15 (3%)	155 (35%)	272 (62%)	
Yes	5 (2%)	60 (27%)	156 (71%)	
Substance abuse				0.04 (2)
No	337 (76%)	88 (20%)	17 (4%)	
Yes	167 (76%)	45 (20%)	9 (4%)	
Leisure/recreation				3.24 (2)
No	31 (7%)	55 (12%)	356 (81%)	
Yes	8 (4%)	26 (12%)	187 (85%)	
Personality/behavior				9.76** (2)
No	182 (41%)	257 (58%)	3 (1%)	
Yes	67 (30%)	149 (67%)	5 (2%)	
Attitudes/orientation				5.46 (2)
No	64 (15%)	360 (81%)	18 (4%)	
Yes	18 (8%)	193 (87%)	10 (5%)	
Overall				19** (2)
No	126 (29%)	270 (61%)	46 (10%)	
Yes	38 (17%)	137 (62%)	46 (21%)	

*Note.* Numbers in parentheses indicate row percentages. YLS/CMI = Youth Level of Service/Case Management Inventory.

** *p* < .01.

## Discussion

This is the first large-scale study examining the relationship between childhood maltreatment and youth offender recidivism in Singapore. With an average follow-up of 4.8 years, the overall recidivism rate in the current sample is 39%, with a range from 14% (within 1 year) to 48% (within 7 years). Comparing with studies conducted in Western countries, the youth offender recidivism rate in Singapore was in the lower range. For example, according to a meta-analysis with 17 studies, the overall mean recidivism rate in Western countries was 48% (ranging from 22% to 75%) with a mean follow-up of 3.8 years (Cottle et al., 2001).

Specifically, the present study showed that the recidivism rate for maltreated offenders was higher at 57% as compared with 38% for nonmaltreated offenders. This pattern of higher recidivism rate among maltreated offenders was similar to previous studies. For example, the recidivism rate was 56% versus 41% among U.S. youth who were involved in both child protection and juvenile probation services as compared with those delinquency cases only (Huang et al., 2012). These results suggest that youth offenders with a childhood maltreatment history are at higher risk of reoffending than those without.

The current study also showed that the maltreated youth offenders had different profiles. For example, the maltreated offenders were arrested for the first time about half a year younger on average than the nonmaltreated offenders. This is consistent with previous research in that childhood maltreatment may “speed up the age” when an individual becomes involved in criminal activities (Widom, 1989). In addition, previous studies have found that earlier engagement in criminal activities is one of the strongest predictors of recidivism (Barrett, Katsiyannis, & Zhang, 2010; Benda, Corwyn, & Toombs, 2001; Cottle et al., 2001; K. C. Thompson & Morris, 2013). Prevention programs can thus focus on dealing with the youth’s early entry into the juvenile justice system, especially for the maltreated children, by promoting effective parenting skills and increasing family supervision (Borum, 2003). It is also important that existing policies relating to youth services ensure that these maltreated youth are promptly provided with the relevant treatment services to address trauma-related problems.

On top of the differences pertaining to personal traits, the current research showed that more maltreated youth offenders were from a background with household and parental problems. These findings suggest that children from certain families are more likely to be maltreated and such differences in their background may confound the true relationship between childhood maltreatment and recidivism. Unlike most of the previous studies that gauge childhood maltreatment effect without a proper control group, the present study used nonmaltreated youth offenders with similar backgrounds as control. Controlling for other variables and differences in the follow-up period, the maltreated youth offenders were 1.38 times as likely to reoffend as the nonmaltreated offenders. In other words, childhood maltreatment was a unique contributor of youth offender recidivism even after controlling for their YLS/CMI 2.0 Overall Risk Ratings, as well as another 15 risk factors relating to their personal characteristics, household environment, and parental background.

There is a possibility that maltreated youth offenders might have developed distinct characteristics that made them more prone to future criminal behavior. Specifically, maltreated youth offenders had significantly higher levels of criminogenic needs in terms of antisocial personality. This suggests that the link between childhood maltreatment and delinquency may be mediated by personality traits. According to a developmental taxonomy theory, a small group of children will engage in antisocial behavior at every life stage whereas a larger group only during adolescence (Moffitt, 1993, 2003). Moreover, children with life-course-persistent antisocial behavior were usually those with neuropsychological problems and such problems might interact with criminogenic environments and eventually lead to pathological personality. Our results seemed to imply that such developmental trajectories for maltreated offenders toward the persistent life-course group. As the current study only examined youth offenders during their adolescence, the life-course hypothesis needs to be tested in studies with longer follow-up period into adulthood.

In addition to the above contribution on theory development, our findings have practical implications on using actuarial risk assessment measures (e.g., the YLS/CMI 2.0) for youth offenders. The unique contribution of childhood maltreatment on recidivism suggests that the YLS/CMI 2.0 Overall Risk Ratings (derived from Singaporean norms) cannot successfully differentiate maltreated offenders, especially in the Low and Moderate Risk category. In fact, the recidivism rates for Maltreated Low Risk and Maltreated Moderate Risk groups were not significantly different from Nonmaltreated High Risk group. Based on these findings, practitioners should be cautious when using the YLS/CMI 2.0 to assess maltreated youth offenders. These findings have important implications for risk classification and management. Notably, these findings could potentially affect the recommendations provided by the probation officers to the courts for their presentence assessments. One recommendation for practitioners is to use professional override for the YLS/CMI 2.0 Overall Risk Rating when assessing maltreated youth offenders. Future studies should focus on the mechanism of recidivism and how to effectively differentiate risk levels for maltreated offenders.

According to the general strain theory, some maltreated children may develop disruptive and delinquent behaviors as a way of coping with stress (Agnew, 1992; Schwalbe, 2008). For example, it was found that delinquent behavior could help a child minimize the negative emotional consequences of strain (Brezina, 1996). In the present study, the maltreated offenders might have come from relatively more impoverished family environments, and had experienced higher levels of strain and frustration. However, the development of maladaptive problem-solving strategies (that were related delinquency) would ultimately contribute to the higher risk of recidivism. Future research is needed to examine this link as it highlights the importance of intervention programs on promoting efficient coping skills as a way to reduce the risk of recidivism.

Our results also emphasize the importance of reducing childhood maltreatment as a central strategy of delinquency prevention. It is thus critical for child protection services to use research-based risk assessment to improve decision making in practice. Such structured assessment of the risk and needs of the childhood maltreatment cases could help reduce subsequent maltreatment and thus benefit not only child protection services but also the juvenile justice system (Wiebush et al., 2001). Moreover, there is evidence to support that childhood maltreatment leads to long-term negative life outcomes (e.g., Allwood & Widom, 2013), and notably, our study also contributes to the extant literature, with more confirmatory evidence for a vicious cycle of reoffending for these maltreated youth offenders. Trauma-informed care that is implemented at systems level could better address the trauma needs and provide meaningful early intervention for youth-at-risk and also for the youth offenders.

One limitation of the study pertains to the measurement of childhood maltreatment. Childhood maltreatment history was coded from the youth offender’s case files; thus, the prevalence of childhood maltreatment might be underestimated. Second, professional override was not used for any case in the current study due to the limitation of retrospective coding of case files. In practice, the practitioners (e.g., probation officers, psychologists, and caseworkers) could have used professional override and provide their own estimate of the risk level based on a more holistic understanding of the youth offenders. Third, the predictive validity might also have been affected by the interrater reliability of ratings for the subscales; although they were not classified as poor, predictive accuracy would most likely be improved with more reliable ratings. Also, gender differences were not examined due to the scope of the study. It is well documented that there are gender differences in the risk factors of recidivism (Benda, 2005; Postlethwait, Barth, & Guo, 2010; Schwalbe, 2008). In other words, the risk assessment tools such as YLS/CMI 2.0 may have differential predictive power for youth offenders with different gender. However, past studies have also shown that childhood maltreatment increases the likelihood of involvement in criminal behaviors for both boys and girls (Widom, 1989). Future studies could examine the interaction effect between gender and maltreatment on the relationship between risk rating and recidivism. Last, PSM derived the matched sample based on the observable information, and the two groups might differ in unobservable ways. Future research should control for more background variables and further reduce the possibility of finding a spurious relationship between child maltreatment and youth offending behavior.

In conclusion, this study demonstrated that youth offenders who had a history of childhood maltreated were more likely to violate their current court orders and commit new criminal offenses. It is possible that the maltreated youth offenders had used delinquent behavior as a way of coping with aversive environment and may gradually develop pathological personality. These results highlight the need for further studies on risk assessment of youth offenders with childhood maltreatment history. Research in this area could better serve children and youth who are dually involved in both child protection and juvenile justice systems.
